# Magnetically guided non-invasive CRISPR-Cas9/gRNA delivery across blood-brain barrier to eradicate latent HIV-1 infection

**DOI:** 10.1038/s41598-019-40222-4

**Published:** 2019-03-08

**Authors:** Ajeet Kaushik, Adriana Yndart, Venkata Atluri, Sneham Tiwari, Asahi Tomitaka, Purnima Gupta, Rahul Dev Jayant, David Alvarez-Carbonell, Kamel Khalili, Madhavan Nair

**Affiliations:** 1 Department of Immunology and Nano-Medicine, Center for Personalized Nanomedicine, Institute of NeuroImmune Pharmacology, Herbert Wertheim College of Medicine, Florida International University, Miami, FL USA; 2 Department of Molecular Biology and Microbiology, Case Western Reserve University, Cleveland, OH USA; 3 Department of Neuroscience, Lewis Katz School of Medicine, Temple University, Philadelphia, 19140 USA

## Abstract

CRISPR-Cas9/gRNA exhibits therapeutic efficacy against latent human immunodeficiency virus (HIV) genome but the delivery of this therapeutic cargo to the brain remains as a challenge. In this research, for the first time, we demonstrated magnetically guided non-invasive delivery of a nano-formulation (NF), composed of Cas9/gRNA bound with magneto-electric nanoparticles (MENPs), across the blood-brain barrier (BBB) to inhibit latent HIV-1 infection in microglial (hμglia)/HIV (HC69) cells. An optimized ac-magnetic field of 60 Oe was applied on NF to release Cas9/gRNA from MENPs surface and to facilitate NF cell uptake resulting in intracellular release and inhibition of HIV. The outcomes suggested that developed NF reduced HIV-LTR expression significantly in comparison to unbound Cas9/gRNA in HIV latent hμglia/HIV (HC69) cells. These findings were also validated qualitatively using fluorescence microscopy to assess NF efficacy against latent HIV in the microglia cells. We believe that CNS delivery of NF (CRISPR/Cas9-gRNA-MENPs) across the BBB certainly will have clinical utility as future personalized nanomedicine to manage neuroHIV/AIDS.

## Introduction

In spite of significant advancements in highly active anti-retroviral therapy (HAART), the elimination of HIV-1 reservoirs from the periphery and central nervous systems (CNS) remains a formidable task^1–7^. HIV-1 undergoes perpetual integration in the host genome, leading to formation of viral reservoirs and also presents a continuous risk of viral reactivation despite the administration of antiretroviral therapy^6,7^. Further, the inability of antiretroviral therapy (ART) to penetrate the blood-brain-barrier (BBB) after systemic administration makes brain as one of the most dominant HIV reservoirs^8,9^. Unfortunately, no effective therapeutics are developed yet to cure neuroHIV. Recent advancements in nanomedicine have shown potential to eradicate HIV via *in-vitro* and *in-vivo* model studies^10–12^. However, this nanomedicine approach, based on developing a nano-formulation (NF) containing an encapsulating biomaterials and multi-component therapeutic agents such as HIV latency breaking agent along with drug to combat against HIV, is tested only at peripheral levels. Beside this, there is a complexity in efficacy and navigation of drugs to CNS due to the strong integrity and semi permeable nature the BBB^13–15^. To address this problem, least component based NFs consisting of a nanocarrier (NCs) and specific therapeutic agent may play a promising role, which can navigate the drug to the CNS for HIV-1 recognition and eradication, thus this becomes an urgent requirement to be explored^8,16^.

Recent introduction of genome editing technology known as clustered regulatory interspaced short palindromic repeat (CRISPR)-associated 9, abbreviated as Cas9, targets integrated HIV-1 within the host genome^4,5,17–23^. Like DNA viruses, HIV can also be targeted by CRISPR/Cas9 manipulation as it has a DNA intermediate in its life cycle. The CRISPR/Cas9 system has two main components: Cas9 (endonuclease) and gRNA which has a 20 bp guide sequence and directs degradation of specific nucleic acids by navigating the gRNA/Cas9 complex to its target by complementary base pairing. Target DNA sequence recognition by Cas9/gRNA requires protospacer adjacent motif (PAM) trinucleotide sequence, which enables targeting and successive endonuclease disruption. Cas9/gRNA together bind to the target DNA sequence, and Cas9 makes a double stranded break (DSB) upstream PAM. These breaks are then repaired by NHEJ (non-homologous end joining) DNA repair mechanism, which is error prone and results in insertions or deletions (InDels) leading to frameshifts or stop codons resulting in disrupted Open Reading Frames (ORFs). Recently, CRISPR/Cas9 is being used to target and eliminate the HIV in latently infected CD4+ T lymphocytic cells, macrophages, and microglia in cell culture, without causing genotoxicity or off-targeting, confirmed by whole-genome sequencing and SURVEYOR assays^17,18^. Yarn like DNA nanoparticles known as DNA nanoclews are the recently discovered vehicle synthesized via rolling circle amplification, and reported as a nanocarrier (NCs) to deliver Cas9/gRNA to the target^24^. Nanoparticle assisted delivery of CRISPR to the brain has recently been demonstrated in mice using intracranial administration to manage fragile X syndrome from exaggerated repetitive behavior^25^. Unfortunately, the non-invasive delivery of Cas9/gRNA across the BBB is not fully explored yet. Site-specific delivery of Cas9/gRNA is extremely promising against retroviruses and more studies in context to HIV are urgently required. Therefore, an effective strategy for controlled delivery of NFs containing Cas9/gRNA across the BBB to activate and eradicate the latent HIV reservoir in the brain is worth exploring.

The one-long-terminal-repeat (1-LTR) circle, the 2-LTR circle, and linear DNA are 3 forms where non-integrated DNA of HIV exists. In HIV-1, Trans-Activator of Transcription (Tat) activates viral transcription by stimulating elongation from LTR. Therefore, LTR will be a target gene for the eradication of latent HIV infection from the body. In this manuscript, we demonstrate a magnetically guided non-invasive delivery and on-demand controlled release of Cas9/gRNA targeting HIV-1 LTR across the BBB using magneto-electric nanoparticles (MENPs) as a drug NCs. The MENPs are ferromagnetic, non-toxic (up to 50 µg), 25 ± 5 nm in size and able to across the BBB under a static magnetic field. The MENPs also exhibited a feature of on-demand release of drug upon external ac-magnetic field stimulation which causes polarization changes on MENPs surface leading to the desired breakdown of bonds between particle and drug. A magnetic NF consisting of Cas9/gRNA bound with MENPs was developed for non-invasive delivery across the BBB under a static magnetic field of 0.8 T. Further, the on-demand release of Cas9/gRNA was achieved on applying ac-magnetic field stimulation via customized electromagnetic coil. The obtained reduced viral LTR expression levels in the Cas9/gRNA treated latent HIV infected hμglia/HIV cells^26^ confirms the successful delivery of NF across the BBB for targeting of latent virus. Such developed therapeutic NF i.e., MENPs-Cas9/gRNA can be used as a potential therapy to target and eradicate latently HIV-1 infection in the brain.

## Results

### NF (MENPs-Cas9/gRNA) preparation

**Figure 1:**
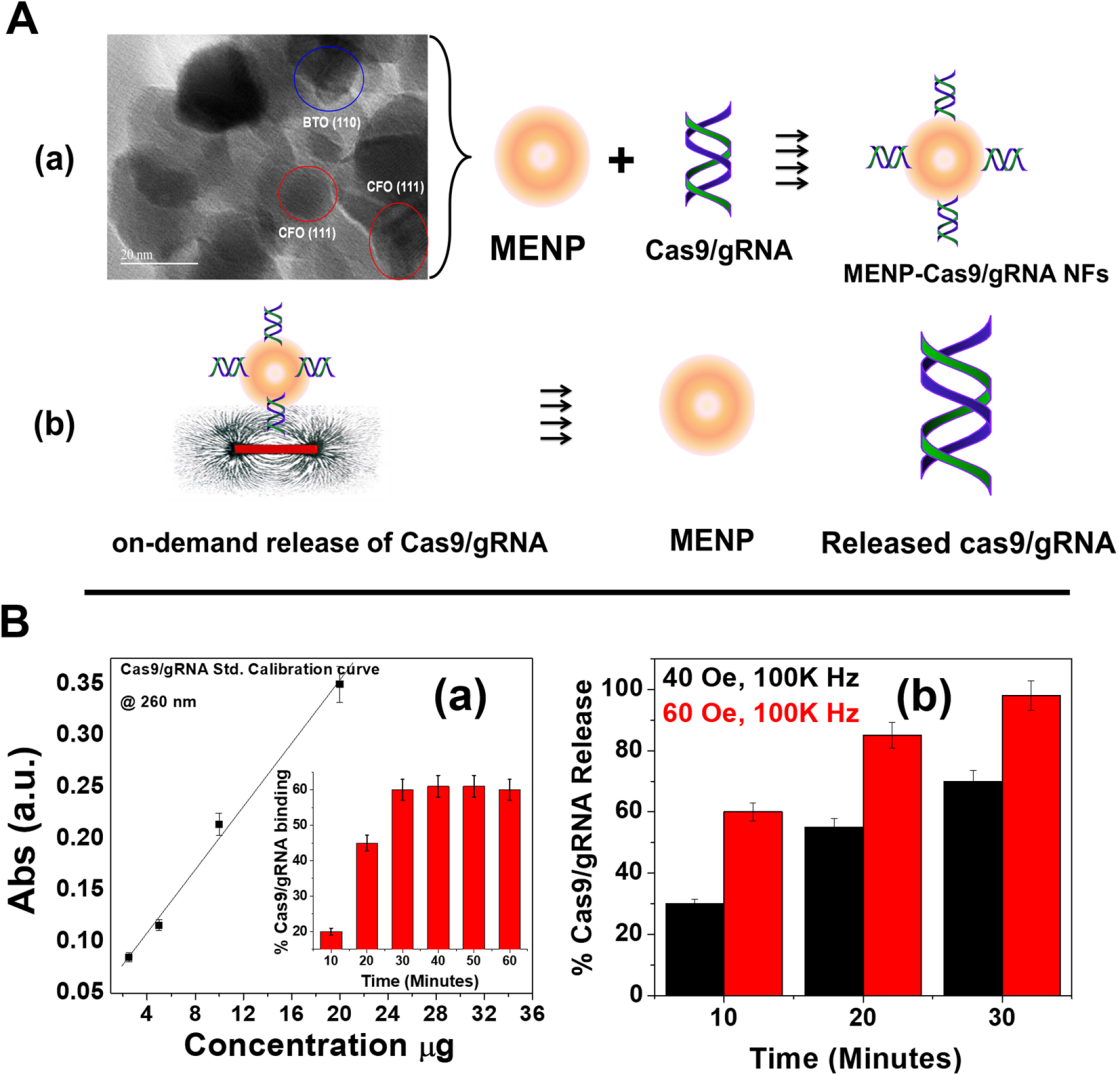
(**A**) Schematic presentation of NF preparation. Surface charged MENPs of size 25 ± 5 nm bind (as demonstrated by representative TEM image) with Cas9/gRNA via electrostatic binding due to differences in surface charge (a). On applying ac-magnetic field stimulation via electromagnetic coil (60 Oe for 30 minutes), the change in polarization on MENPs causes MENPs-Cas9/gRNA bond length contraction and expansion. This rapid phenomena occurred repeatedly and at some points, the bond between MENPs and Cas9/gRNA broke down. (**B**) Cas9/gRNA and binding and release profile. (a) Calibration curve plotted between Cas9/gRNA absorption at 260 nm as a function of Cas9/gRNA concentrations. This calibration curve was used to estimate unknown concentration of Cas9/gRNA. Cas9/gRNA % binding with MENPs (50 µg) as a function of time is shown in inset (a). (b) On-demand release of Cas9/gRNA from MENPs surface as a function of externally applied ac-magnetic field and time at constant frequency (1 KHz).

The MENPs utilized in this research as a Cas9/gRNA carriers are well characterized using a vibrating-sample magnetometer (VSM) for magnetization assessment, transmission electron microscopy (TEM) for particles size estimation, X-ray diffraction (XRD) pattern for phase purity evaluation. The outcomes of these studies have been described in our previous manuscript^16,27^. In brief, MENPs [BaTiO_3_ (BTO) @CoFe_2_O_4_ (CFO)] of particle size ranging from 20 to 30 nm (Fig. 1Aa) exhibited diffraction peaks associated with crystallographic planes of CFO (JCPDS 00-022-1086) and BTO (JCPDS 04-001-7269) confirming the phase purity and crystalline characteristics of MENPs composed of BTO and CFO phases^16,27^. This arrangement of magnetic core and piezoelectric shell showed a ferromagnetic magnetism (34 emu/g), a desired property of an electro-active material to achieve on-demand release of a drug from the MENPs surface system through an externally applied ac-magnetic field stimulation^27^.

The MENPs are monodispersed (PDI value of 0.24) positively surface charged (zeta potential −30 mV) nanoparticles and can easily bind with negatively charged Cas9/gRNA system via electrostatic interaction. This low value of PDI reveals mono-dispersive characteristics of MENPs useful for good binding with water soluble drugs like Cas9/gRNA and systemic administration in targeted animal model^16^. The strategy of MENPs-Cas9/gRNA NF preparation and Cas9/gRNA release on ac-magnetic field stimulation is illustrated in Fig. 1A and described with scientific reasoning in respective sections. Time kinetics of Cas9/gRNA binding with MENPs and further release profile of cas9/gRNA from NF as a function of ac-magnetic field stimulation were studied using optical density (OD) method (Fig. 1B). The bound Cas9/gRNA concentration was estimated via measuring OD (at 260 nm) of unbound fraction of plasmid (supernatant). Based on findings of OD study, a calibration curve was established as a function of absorption intensity at 260 nm and Cas9/gRNA concentrations ranging from 1 to 20 µg (Fig. 1Ba). This linear (r^2^ = 0.989) calibration curve was used to estimate % binding of Cas9/gRNA over the time and % release of Cas9/gRNA as a function of applied magnetic field. The results of OD density showed that 60% of 10 µg Cas9/gRNA binds with 50 µg of MENPs within 30 minutes of incubation time (Inset, Fig. 1Ba). Since, only 60% cas9/gRNA bind with MENPs, we selected only 6 µg of Cas9/gRNA to prepare NF to conduct further experiments.

The NF i.e. MENPs-Cas9/gRNA was exposed to an ac-magnetic field generated from an indigenously customized electromagnetic coils. Externally applied ac-magnetic fields stimulation (40 and 60 Oe at 100 kHz) exposed to NF alter polarization in MENPs and cause expansion and contraction in ionic bonds available on MENPs surface. This phenomena occurs repeatedly and break electrostatic bonding and releases Cas9/gRNA system from MENPs surface. After applying ac-magnetic field, the mixture was centrifuged to conduct OD study for the estimation of % release Cas9/gRNA system. The results of OD suggested that the release of Cas9/gRNA system is a function of applied field and stimulation time. At a constant exposure time for 30 minutes, applied filed of 40 Oe field exhibited 70% release of Cas9/gRNA, while approximately 98% Cas9/gRNA release was obtained on applying magnetic field of 60 Oe (Fig. 1Bb). These findings suggest that a magnetic field of 60 Oe applied for 30 minutes would be enough to release maximum % of plasmid in *in-vitro* BBB models also.

### Characterization MENPs-Cas9/gRNA formulation

**Figure 2:**
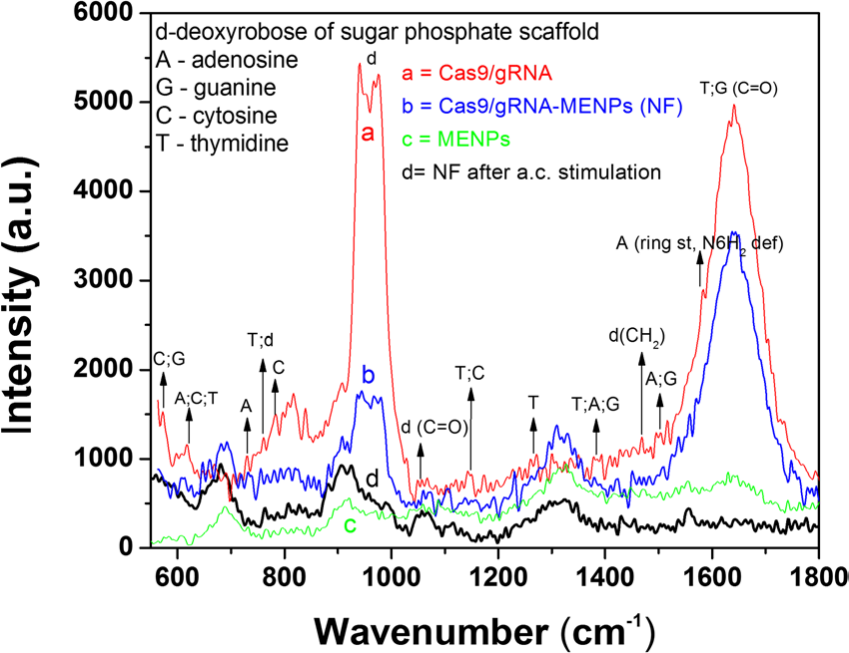
Qualitative confirmation of NF preparation and the release of Cas9/gRNA using Raman spectroscopy. Raman spectra of Cas9/gRNA (curve a), MENPs-Cas9/gRNA NF (curve b), MENPs (curve c) NF after external ac-magnetic field stimulation (curve d).

The qualitative assessment of the possible formation and deformation of binding during preparation of MENPs-Cas9/gRNA NF and on-demand release of Cas9/gRNA from NF were evaluated using Raman spectroscopy (Fig. 2). Nucleic acids exhibits strong Raman bands in mid-frequency region (600 to 1800 cm^−1^) corresponds to intramolecular modes of nucleic acid bases such as adenosine (A), guanine (G), cytosine (C), thymidine (T), d-deoxyribose of sugar phosphate scaffold. We also selected this region to evaluate NF preparation and release of Cas9/gRNA. The Raman spectra of Cas9/gRNA (curve a) exhibited all optically active modes corresponds to the bases alone and in combination i.e., A, G, C, & T [570 cm-1 (C;G), 621 cm^−1^ (A;C;T), 630 cm^−1^ (A), 760 cm^−1^ (T;d), 780 cm^−1^ (C), 1150 cm^−1^ (T;C), 1380 cm^−1^ (T;A;G), 1505 cm^−1^ (A;G), and 1580 cm^−1^ (A ring stretching, N6H_2_ deformation)]^28,29^ present in nucleic acid chain, DNA backbone (800 to 1000 cm^−1^)^30^, and optically active modes related with d at 900 to 1000 cm^−1^ (sharp bands of higher intensity), d (C=O) at 1050 cm^−1^ and d (CH_2_) at 1500 cm^−1^ as shown in curve a. On comparative Raman analysis of NF (curve b), we observed that NF consisting MENPs-Cas9/gRNA exhibited all possible band of modes related with Cas9/gRNA (curve a) and MENPs (curve c). The peak position of Raman modes in NF exhibits a small shift due to ionic bond formation between MENPs and Cas9/gRNA via electrostatic interaction.

The MENPs exhibited Raman active modes (curve c) associated with BTO^31^ and CFO^32^ to confirm the presence of MENPs i.e., BTO@CFO. Raman mode at 500 cm^−1^ and 670 cm^−1^ are attributed to tetragonal phase of BTO and cubical inverse-spinel structure, respectively. Raman observed below 300 cm^−1^ is attributed to E (transversal (TO) and longitudinal (LO) components TO + LO) mixed mode. Raman band at 515 cm^−1^ corresponds to transversal modes i.e., E (TO) and A_1_ (TO) of BTO^31^. Further Raman peak around 800 cm^−1^ corresponds to stacking-fault density of BTO phase, and appears due to high temperature calcination of MENPs at 780 °C. The cubic spinal active Raman modes, mainly due to E_g_, and three of F_2g_ symmetries were observed at 300 cm^−1^. The peak observed at 670 cm^−1^ is attributed to the F_2g_ symmetry of CFO which is a characteristic Co-O displacement.

The NF was stimulated through ac-magnetic field and as explained above, it detached Cas9/gRNA from MENPs surface. Raman spectra of NF after ac-magnetic field stimulation was measured (curve d) to prove the release of Cas9/gRNA through breaking of ionic bonds on ac-magnetic field stimulation. It was observed that Raman modes associated with Cas9/gRNA disappeared, which confirm the release of Cas/gRNA. Though 100% drug release in general is not possible, this is the reason that some bands of low intensities are still visible in curve. However, the Raman modes observed in NF after ac-magnetic field stimulation seem corresponds to MENPs only. This confirmed the release of Cas9/gRNA from NF due to breakdown of bonding through ac-magnetic field stimulation via electromagnetic coil.

### Toxicity assessment of MENPs and NFs (MENPs-Cas9/gRNA)

**Figure 3:**
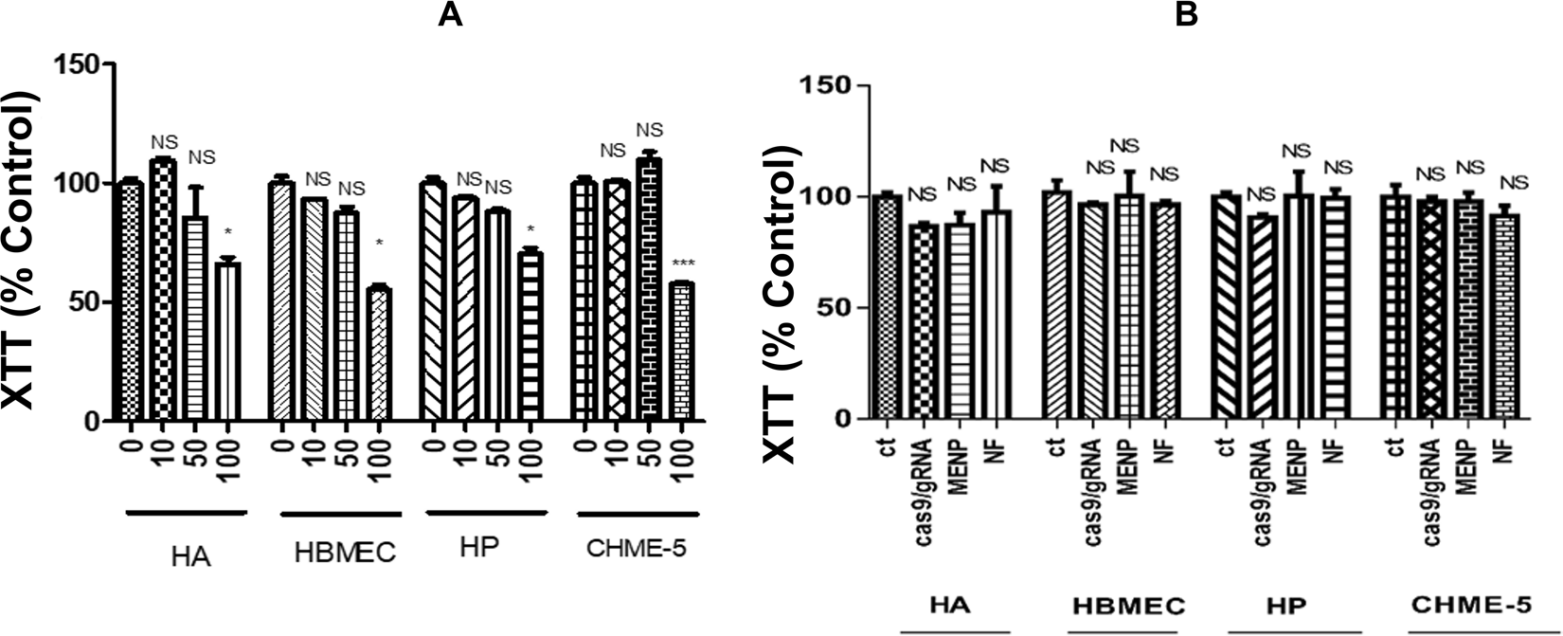
Cytotoxicity study of MENPs and NF with respect to CNS cell tyoes (HA, HP, HBMECs, and CHEM-5). (**A**) XTT assay of MENPs concertation ranging from 10 to 1000 µg, (**B**) XTT assay of MENPs (50 µg), Cas9/gRNA (6 µg), and NF [MENPs (50 µg)-Cas9/gRNA (6 µg)] to assess CNS cells viability. (*p ≤ 0.05; ***p ≤ 0.001; NS-Not Significant).

The *in-vitro* cytotoxicity of MENPs (10–100 µg), Cas9/gRNA (6 µg) and NF [MENPs (50 µg)-Cas9/gRNA (6 µg)] was assessed by performing XTT assay (Fig. 3). Cells associated with *in-vitro* BBB model; Human Astrocytes (HA), Human Pericytes (HP), Human Brain Microvascular Endothelial Cells (HBMEC), and CHME5 cell lines upon treatment with MENPs concentrations ranging from 10 to 50 µg exhibited approximately 90% cell viability compared to control (Fig. 3A). However, cell viability reduced to 70% upon treatment with high dose of MENPs (100 µg). Therefore 50 µg of MENPs was found to be an optimized dose for preparing a NF using Cas9/gRNA system (6 µg). The MENP concentrations we have used in this cytotoxicity experiment are very high considering the number of cells we have used, the surface area and amount of the media. We have chosen to use these broad ranges of MENP concentrations to determine the biosafety of our nanoformulations even at high concentrations. We have observed no cytotoxicity up to 50 µg of MENP which indicate the biosafety of our nanoparticles even at high concentrations.

The XTT assay performed on CNS cells (HA, HP, HBMEC, CHME5) treated with MENPs (50 µg), Cas9/gRNA (6 µg) and NF (MENPs-Cas9/gRNA formulation) (Fig. 3B) showed cell viability of around 90% which indicates no toxic effects during the treatments. This implies that our prepared MENPs-Cas9/gRNA NF is safe for cells *in-vitro* and can be explored for further use *in-vivo* systems.

### Evaluation of *in-vitro* BBB integrity and NF transmigration across the BBB

**Figure 4:**
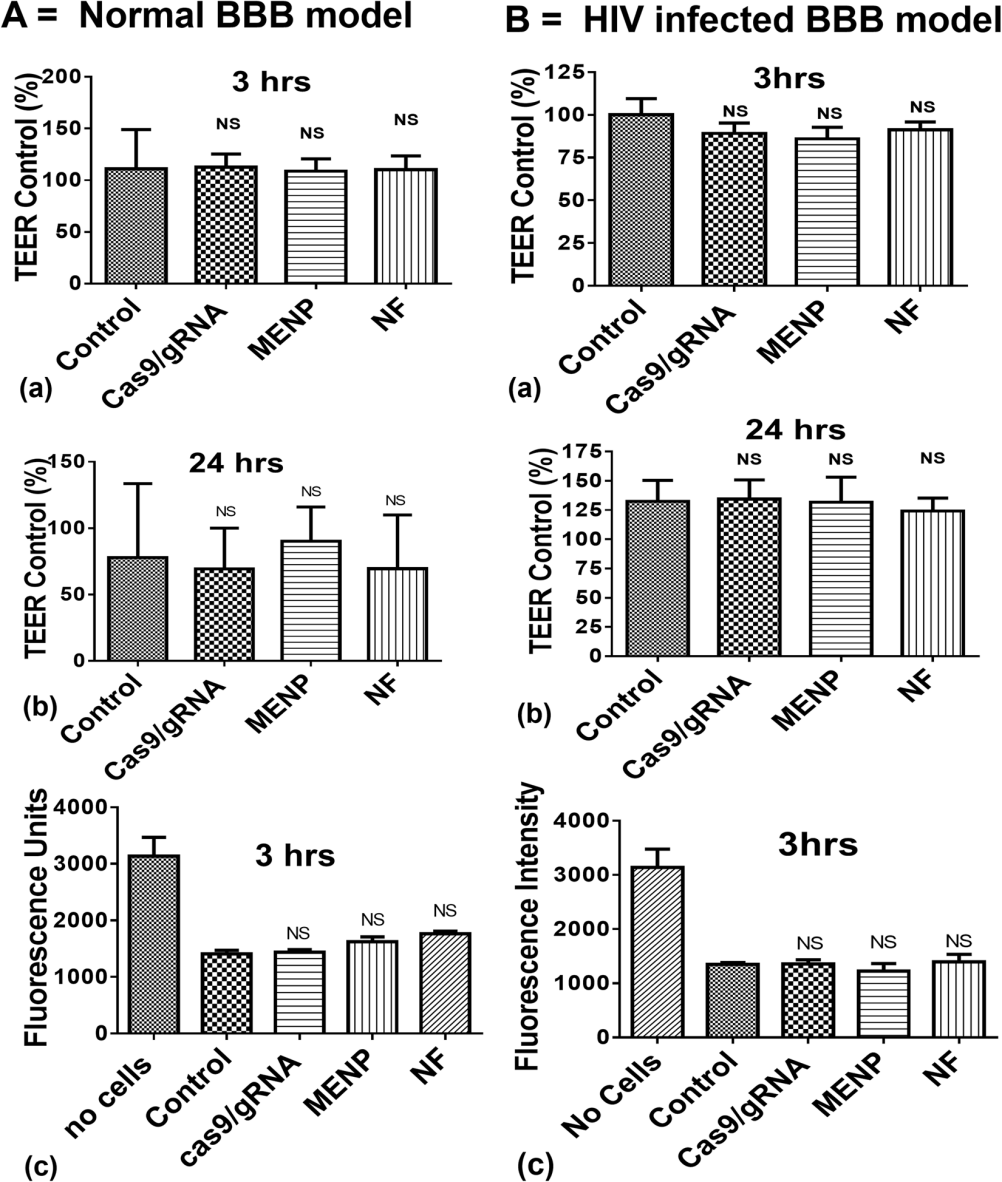
Effect of MENPs (50 µg), Cas9/gRNA (6 µg), and NF [MENPs (50 µg)-Cas9/gRNA (6 µg)] on BBB integrity (**A**) and HIV infected (**B**) *in-vitro* model. Magnetically guided navigation of MENPs-Cas9/gRNA NFs. An optimized dose of NF consist of MENPs (50 µg) and Cas9/gRNA (6 µg) was spread on top section of BBB for navigation across BBB under a static magnetic field (0.8 T for 3 hrs). (Ns-Not significant).

Various parameters such as nanoparticle at interface of cells, HIV infection, and magnetic field stimulation may affect the tight junction of BBB and facilitate the transmigration of unwanted element to the brain. Thus exploring BBB junction integrity becomes crucial in this study. An *in-vitro* BBB model was placed on a static magnet (0.8 T) for 3 hours to facilitate navigation of NF across the BBB as illustrated in Fig. 4A. The upper chambers of the both normal (Fig. 4A) and HIV infected (Fig. 4B) BBB models, were treated with MENPs (50 µg), Cas9/gRNA (6 µg) and NF. The integrity of BBB model was evaluated via measuring transepithelial electrical resistance (TEER) values and Fluorescein isothiocyanate (FITC) intensity of control and each treated wells. The findings from TEER values and FITC experiments explained that each treatments does not affect BBB integration in the both models.

Ammonium thiocyanate-based photometric assay (490 nm) was performed to estimate concentration of MENPs which was able to cross the BBB. The assay results suggested that 26% MENPs crossed the BBB *in-vitro*. Upon estimation of OD, from media collected from the lower chamber of the BBB model after ac-magnetic field stimulation, we observed that that 2 µg Cas9/gRNA was released in the lower chamber subsequent to the BBB transmigration.

### On-demand release and functionality evaluation of Cas9/gRNA system

**Figure 5:**
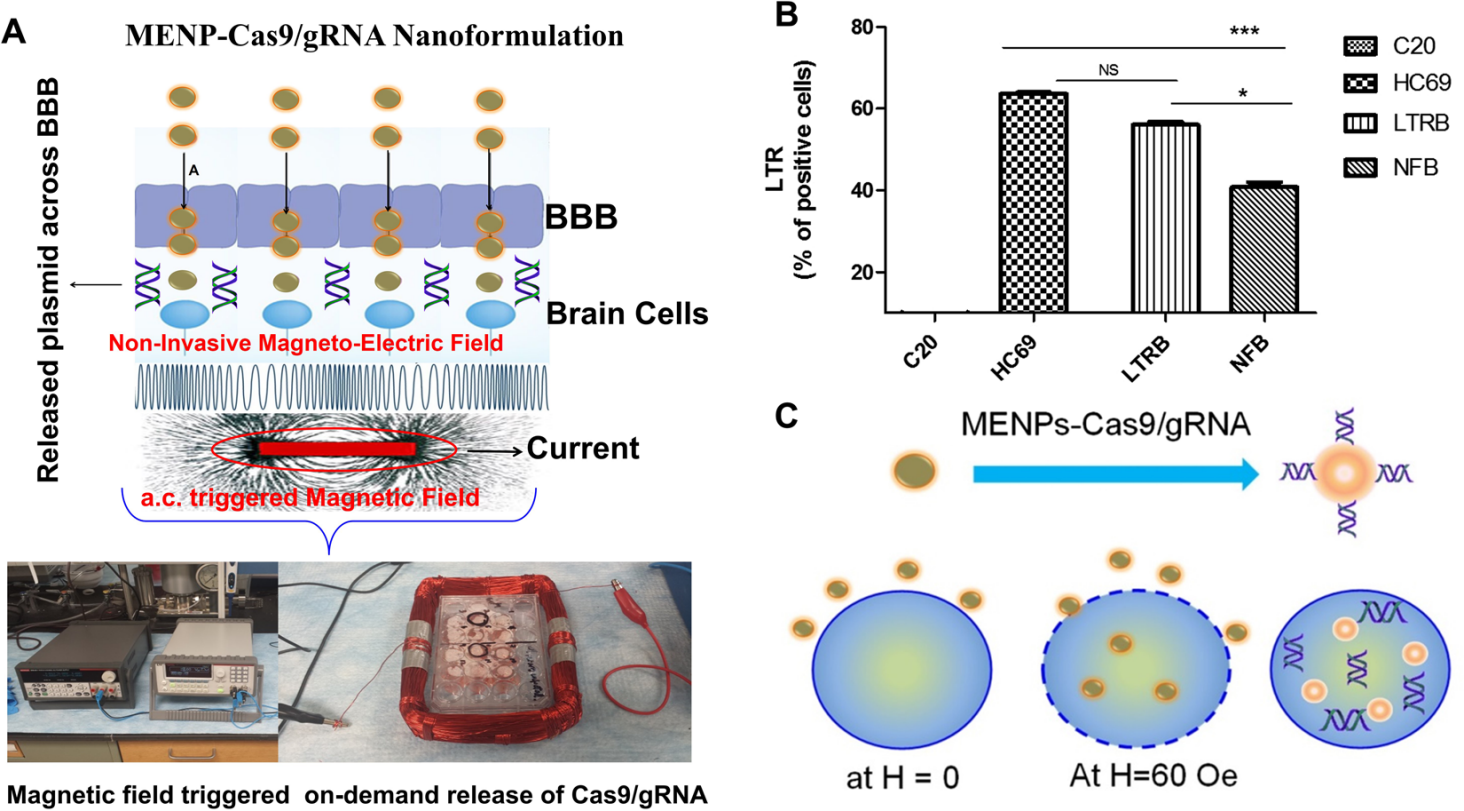
(**A**) On-demand release of Cas9/gRNA form MENPs surface and plasmid efficacy assessment. An optimized ac-magnetic field (60 Oe for 30 minutes) was applied through an electromagnetic coil to generate enough stimulation to break the bond between Cas9/gRNA and MENP. (**B**) Bio-functional activity of released Cas9/gRNA will eradicate latently infected HIV reservoirs at cellular level, and (**C**) an illustration of NF up taken by MG cells via nano-electro-poration. (*p ≤ 0.05; ***p ≤ 0.001; NS-Not Significant).

To release Cas9/gRNA from NF, HIV-infected BBB model was placed in an electromagnetic coil for ac-magnetic stimulation, as illustrated in Fig. 5A. An optimized ac-magnetic field of 60 Oe was applied for 30 minutes to achieve Cas9/gRNA release in the lower chamber of the BBB model. To compare the functionality of released Cas9/gRNA, an identical experiment was performed wherein, the BBB was treated using only Cas9/gRNA. The media from lower chamber of treated BBB model (with HIV alone, Cas9/gRNA-HIV, and NF-HIV) was collected and amount of released Cas9/gRNA was estimated, by deciphering it ability to reduce HIV long terminal repeat (LTR) expression level (Fig. 5B) in HC69 cells grown in basal chamber of the *in vitro* BBB model.

The LTR level in HIV infected sample was estimated as 65, compared to uninfected control. In case of only Cas9/gRNA treated the BBB, the treatment reduced LTR level to 48 confirming that Cas9/gRNA remains functional and does not lose its activity on applying ac-magnetic field. The LTR expression levels reduced remarkably to 40 on treatment with released Cas9/gRNA from NF on ac-magnetic field stimulation. This significant reduction in LTR expression, compared to HIV infected cells, may be attributed to intracellular eradicating mechanism due to significantly high cell uptake.

This is due to easy and high cell uptake of NF via electroporation on applying ac-magnetic field. Electroporation in cells on applying ac-magnetic field of 60 Oe has been demonstrated in our previous publication^12^. In brief, CFO core of MENPs went through a strain change on applying ac-magnetic field stimulation. This deformation was further absorbed by BTO piezoelectric shell of MENPs to cause pressure waves. This generated pressure wave is capable to misaligned phospholipid later of cell member to cause poration when MENPs is at µm distance from the cell. This ac-magnetic field induced nano-electro-poration caused NF delivery inside the cell resulted in higher efficacy.

**Figure 6:**
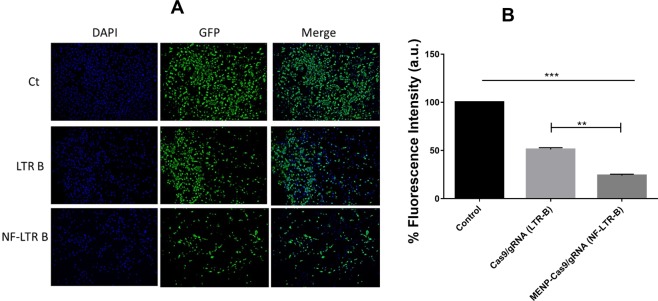
Immunofluorescence analysis of HC69.5 cells on treatment of LTR-B plasmid B and NF. Clone HC69.5 (HIV/GFP) were treated with LTR-B plasmid, and MENP/LTR-B plasmids NF. Cells were cultured under 1% of FBS and incubated for 2 hrs after treatment. After the release procedure, cells were fixed using PFA 4% and visualized using Keyence ALL in one microscope (10X). (**p ≤ 0.01; ***p ≤ 0.001; NS-Not Significant).

Due to electroporation, there is a high uptake of NF by H-MG cells followed up by intracellular release of Cas9/gRNA on stimulation, as schematically illustrated in Fig. 5C. This phenomena cause HIV-infection eradication intracellularly resulting in more reduction in HIV-LTR expression level than using pure Cas9/gRNA. In the quantitative analysis of the efficacy of Cas9/gRNA and its MENP based NF using florescent microscopy (Fig. 6A), we have observed significantly reduced GFP fluorescence in Cas9/gRNA treated cells when compared to the untreated control cells indicating significantly reduced latent infection in HIV-GFP cells. Furthermore, using MENP based NF of Cas9/gRNA, we have observed further reduction of latent infection when compared to the Cas9/gRNA alone treatment. Intensity of the florescence is directly proportional to the HIV-1 LTR levels which is an indicative of HIV infection levels in those cells. Quantitative assessment of fluorescent intensity analysis of images indicates that NF is more efficient in reducing the latent infection levels when compared to the Cas9/gRNA alone (Fig. 6B).

## Discussion

We propose positively surface charged mono-dispersed ferromagnetic MENPs as potential NCs to navigate Cas9/gRNA across the BBB under the influence of static magnetic field, which is safe and non-invasive. Additionally, we demonstrated an on-demand release of Cas9/gRNA from NF on applying ac-magnetic field externally through electromagnetic coil. The Cas9/gRNA binds to MENPs via electrostatic binding due to opposite surface charges, as demonstrated by Raman analysis. The results of OD studies values demonstrated that within 30 minutes of incubation time, 60% Cas9/gRNA of 10 µg with 50 µg of MENPs and ~98% Cas9/gRNA released on applying 60 Oe ac-magnetic field. The cytotoxicity studies and TEER value measurement studies confirmed that MENPs (50 µg), Cas9/gRNA system (6 µg) and NF do not adversely affect cell viability and also the BBB integrity on each respective treatment. Our developed NF has the ability to cross the BBB, *in-vitro* model, under the influence of a static magnetic field due to the magnetic nature of NF. Thus demonstrated magnetically guided non-invasive BBB transmigration technique may be effective and practicable over the conventional techniques such as focused ultrasound methods, electric field, photosensitization etc. These methods are not well adopted due to transient BBB opening which can allow entrance of other foreign moieties^8,33^ which induce cell damage and slow recovery. We estimated that 26% MENPs navigated across the BBB which is significant in comparison of TAT-mediated^34^ and liposome^35^ encapsulated delivery of organic and inorganic nanostructures for drug delivery application. Development of an efficient NF which can be guided externally across the BBB without any side effects becomes extremely important. Thus our proposed delivery method seems to be one of the potential solution to overcome challenges^8^.

Cas9/gRNA was released across the BBB, in controlled manner via applying an optimized ac-magnetic field of 60 Oe for 30 min. The released Cas9/gRNA was biologically active and reduced HIV-LTR expression levels in latent HIV-1 infected cells. The results of presented studies showed that the Cas9/gRNA released form NF exhibit superior therapeutic efficacy in comparison of treatment using Cas9/gRNA only. This is attributed to higher uptake of NF due to electroporation and its ability to target latent HIV-1 genome suggesting as a promising therapeutic approach. Our investigated on-demand and controlled intracellular HIV-1 infection eradication approach using nanotechnology is unique and has never been demonstrated before. The other available approaches are multicomponent therapeutics to activate latent virus and further to eradicate HIV infection. The integration of all these agents results in a large size (>150) which exhibit poor efficacy because of size constraints due to intact integrity of the BBB^36,37^. The development of effective therapeutic NFs based on minimal sized therapeutic agent system which can cross the BBB and activate and eradicate HIV in brain reservoirs are urgently required. We demonstrate that designed Cas9/gRNA recognize and eliminate HIV-1 infection, confirmed by reduction in LTR expression level, in CNS cells upon successful delivery across the BBB using nanotechnology with the feature of on-demand release and no noticeable side-effects. This technology is of crucial significance against HIV reservoirs in the brain. Earlier studies by Dr. Khalili’s group recently reported that Cas9/gRNA specifically targeting U3 region of HIV-1 LTR, inactivates viral gene transcription and thereby the viral replication in latently infected cells^38^. Our results suggest that MENP guided delivery of Cas9/gRNA across the BBB will have a translational significance in eradicating HIV latency in the brain.

In conclusion, we demonstrated non-invasive magnetically guided delivery of Cas9/gRNA across the BBB using MENPs as a drug NC. High cell-uptake of NF via nano-electroporation and on-demand release of Cas9/gRNA (98%) through ac-magnetic field stimulation resulting in significant HIV-LTR reduction than control Cas9/gRNA. Due to salient features such as bio-compatibility, non-invasive CNS delivery, and on-demand Cas9/gRNA release our developed NF has potential to manage neuroHIV. As a viewpoint, aiming to develop a Cas9/gRNA nanomedicine as therapy for neuroHIV, this such developed NF delivery need to be demonstrated in appropriate animal model. Authors are in process to optimized/develop an appropriate animal to demonstrate herein proposed technology to eradicate neuroHIV. We have previously demonstrated non-invasive and safe delivery of MENPs to the mice brain and observed uniform distribution of MENPs in all brain cell types without agglomeration and without altering motor coordination function of mice^16^. As future direction, in combination with our established magnetically guided delivery to the brain technology, Cas9/gRNA can successfully be delivered to the brain for the eradication of neuroHIV/AIDS.

### Online methods

#### Cas9/gRNA selection

A complete procedure, bio-informatics screening, genetic sequences, functionality and capability to reduce HIV-LTR of CRISPR designed Cas9/gRNA employed in this work has been discussed by Khalili *et al*.^38^. Bio-informatics screening and efficiency/off target prediction identified four gRNA targets within the HIV-1 LTR, named A, B, C and D that avoid conserved transcription factor binding sites, minimizing the likelihood of altering host gene expression. Expression of these gRNAs by a vector that also expresses the Cas9 protein in the CHME5 cells, a microglial cell line that harbors integrated copies of a single round HIV-1 reporter (pNL4-3-ΔGag-d2EGFP), drastically suppressed LTR promoter-driven expression as measured by the fraction of EGFP positive cells, reported in previous publication^38^. The gRNA specific to LTR-B in CHME-5 cells exhibited maximum reduction in LTR promoter-driven expression shown by p24 and LTR fold change. We have used Cas9/gRNA specific to LTR-B for navigation across the BBB using MENPs as NCs non-invasively. Dr. Khalili’s group has reported at the multiplex expression of LTR A/B gRNAs in CHME5 cells caused deletion of 190 bp fragment between the A & B target sites and led to junction site mutations to various degrees as detected by PCR and Sanger sequencing^38^. This treatment completely suppressed the appearance of EGFP positive cells, due to inhibition of LTR transcription in the cells. This group has estimated viral load using p24 ELISA and real time PCR analysis. The results of these studies showed a substantial decrease in viral replication. The low, but detectable, residual viral presence is almost certainly due to the background of transfected cells.

#### Synthesis of MENPs

The MENPs of BaTiO_3_@CoFe_2_O_4,_ composed of two precursors namely CoFe_2_O_4_ (CFO) and BaTiO_3_ (BTO) are synthesized using our established three steps method^11,12^. The synthesize CFO nanoparticles 15 mL of aqueous mixture of 0.058 g of Co(NO_3_)_2_.6H_2_O, 0.16 g of Fe(NO_3_)_3_.9H_2_O and 0.2 g of polyvinylpyrrolidone (Av. Mol. Wt. −40,000) is dissolved in 5 mL of aqueous 0.9 g of sodium borohydride at 120 °C for 12 h. The BTO precursor solution was prepared by mixing two solutions a) 0.029 g of BaCO_3_, 0.1 g of citric acid with 30 mL of ethanol solution and b) 0.048 mL titanium isopropoxide and 1 g of citric acid are mixed to prepare BaTiO_3_ precursor solution. Finally, MENPs were prepared by dispersing 0.1 g of CoFe_2_O_4_ nanoparticles in the BTO precursor solution under sonication for 2 h, further dried at 60 °C for overnight, and followed by calcination at 780 °C for 5 h. Synthesized MENPs were characterized FEI CM 200 transmission electron microscope (TEM), X-ray Diffractometer (XRD, based on Mo-Kα radiation) to evaluate morphology, particle size, and crystalline nature. Energy dispersive spectroscopy (EDS) and vibrational sample magnetometer (VSM) studies were also conducted to evaluate chemical composition and magnetic behavior of MENPs.

#### Fabrication and characterization of MENPs-Cas9/gRNA NF

To prepare a NF, 10 µg of Cas9/gRNA was dispersed in 100 µL (PBS) containing 50 µg of MENPs in an Eppendorf on rotating platform. After 30 minutes, mixture was centrifuged at 2000 rpm for 2 minute. The supernatant containing MENPs bound Cas9/gRNA was collected and washed with PBS (pH 7.4) to remove unbound Cas9/gRNA. The upper part of solution contains only unbound Cas9/gRNA which was further quantified to know the amount of bound plasmid with reference to control. To determine time kinetics, a calibration curve of Cag9/gRNA was established as a function of known concentration and absorption intensity at 280 nm. Thus established calibration curve was used to estimate bound or percentage binding of Cas9/gRNA with MENPs. To explore time kinetics, percentage binding of Cas9/gRNA (10 µg) with MENPs (50 µg) was studied as a function of time (10 to 30 minutes). The stepwise preparation of MENPs-Cas9/gRNA NF was characterized using Raman spectroscopy (Nomadic Raman microscope with BaySpec 532 nm laser) to confirm possible bindings between precursors. 20 µL of cas9/gRNA (10 µg), MENPs (50 µg), and MENPs-Cas9/gRNA (50 µg:10 µg) solution in DI water was drop-casted on silica substrate to perform Raman Spectroscopy.

#### Measurement of cytotoxicity

The XTT assay was performed to evaluate toxicity of MENPs (10 to 100 µg), Cas9/gRNA (1 to 10 µg) and MENPs-Cas9/gRNA (50 µg: 6 µg) NF in CNS cell lines used in *in-vitro* BBB model (Human Brain Microvascular Endothelial Cells (HBMEC, Cat# 1000; Sciencell); Human Astrocytes (HA, Cat# 1800; Sciencell); Human Brain Vascular Pericytes (HP, Cat# 1200; Sciencell) and Microglia cells (CHME-5, obtained from *Dr. Jose Rodriguez, Universidad Central del Caribe, Puerto Rico*)). The CNS cell types were assayed for cell proliferation and viability by XTT assay (Cat #30-1011 K; ATCC) after 4 hours and 24 hours of treatment, in order to investigate the cytotoxicity of the different concentration of MENPs. Populations of 1 × 10^4^ cells were seeded in 96 well plate and cells were treated with different concentration of MENPs, Cas9/gRNA and NF. The XTT assay was measured at 475 nm and 660 nm using Biotek Synergy HT multimode microplate reader instrument. The results were expressed as percentage of cell viability compared to control cells.

#### Preparation of *in-vitro* BBB model and navigation of NF across the BBB

An established procedure as described earlier was used to prepare a BBB model^39^. The model consisted of two-compartment wells in a culture plate, with the upper compartment separated from the lower by a cyclopore polyethylene terephthalate membrane (Collaborative Biochemical Products, Becton Dickinson, San Jose, CA) with a pore diameter of 3 μm. In a 24 well cell culture insert, 2 × 10^5^ primary HBMECs were grown to confluency on the upper side whereas a confluent layer of primary HA and HP (1 × 10^5^ cells/insert) were grown on the underside. A 0.5 × 10^5^ CHME-5 cells population was added to the 24 well plate surfaces. Intactness of the BBB was determined by measuring the transendothelial electrical resistance (TEER) using Millicell ERS microelectrodes (Millipore, Billerica, MA). To prepare a HIV infected the BBB model, CHME-5 HIV infected cells (5dpi) were added to the lower surface of the 24 well plate after checking the intactness of the BBB. The BBB model was used for experiments at least 5 days after cell seeding.

The upper chamber of such constructed BBB was treated with 100 µL of MENPs (50 µg), Cas9/gRNA (10 µg), and MENPs-Cas9/gRNA (50 µg: 6 µg) NF. BBB containing 24 well plate was placed on top of a static magnet (0.8 T) for 3 hour. The applied static magnetic field facilitates transmigration of MENPs containing NF across the BBB^10^. TEER measurements were performed after 3 hrs and 24 hrs of treatments. The electrical resistance of blank inserts with medium alone was subtracted from TEER readings obtained from inserts with confluent monolayers. The resulting TEER values represent the resistance of the endothelial cell monolayers. The results are presented as percent of control. In parallel, the effects of the treatment on the paracellular transport of flourescein isothiocyanate–labeled (FITC) dextran (molecular weight 40000) were measured according to the procedure described earlier^40,41^. FITC-dextran (Sigma Aldrich) of 100 mg/mL was added to the upper chamber of the inserts and further incubated for 4 h. Samples were collected from the bottom chamber after 4 h and fluorescence intensity was measured using a excitation wavelength of 485 nm and emission wavelength of 520 nm using Biotek Synergy HT multimode microplate reader instrument. FITC-dextran transport was expressed as fluorescence units of FITC-dextran transported across the BBB into the lower compartment. All data are presented as the mean ± the standard deviation of the mean (S.D). The results were analyzed using ANOVA (Kruskal-Wallis test) followed by Dunn’s Multiple Comparison post- test to determine statistical significance (Graph Pad 5 Software, Inc., La Jolla, CA, USA).

The navigation of MENPs across the BBB to the lower chamber was quantitatively confirmed via estimating Fe ion concentration using ammonium thiocyanate-based photometry assay as described in our previous paper^10^. The qualitative confirmation of MENPs was also performed using TEM imaging of lower chamber media.

#### On-demand release of Cas9/gRNA and functional assessment

A 24 well-plate containing MENPs-Cas9/gRNA NF navigated across the BBB was placed in an indigenously designed electromagnetic coil (dimension 4 × 6 inches) for field control release of Cas9/gRNA system. The Cas9/gRNA release procedure was performed using ac-magnetic field of 40 and 60 Oe at a constant frequency of 1 KHz and time of 30 minutes to optimize maximum drug release (around 98%). These H-MG and HIV latent H-MG cells were provided by the research group of Dr. Jonathan Karn, Department of Molecular Biology and Microbiology, Case Western Reserve University, Cleveland, OH.

HC69.5 (HIV/GFP) and HIV-GFP cells were treated with LTR-B plasmid (Cas9/gRNA) and related MENPs based NF. Cells were incubated for 2 hrs followed by applying ac-magnetic field (60 Oe for 30 minutes) to release the plasmid. The functionality assessment of release plasmid was performed via LTR expression (quantitative) and immunofluorescence imaging (qualitative) as a function of GFP fluorescence. For LTR expression, cells were cultured under 1% of FBS. The C20 clone, the parenteral cells were used as a negative control. RNA was extracted using RNeasy Mini kit (QIAGEN) and reverse transcribed (High-Capacity cDNA Reverse Transcription Kit; Life technologies) followed by quantitative real time analysis using PCR for LTR gene. For fluorescence microscopic analysis, clone HC69.5 (HIV/GFP) cells treated with LTR-B (Cas9/gRNA) plasmid and related NF was cultured under 1% of FBS and incubated for 2hrs followed by applying release conditions identically. After this procedure, the cells were fixed using PFA 4% to perform fluorescence imaging using a Keyence ALL in one microscope (10X)

### Statistical analysis

Experiments conducted in this study were performed at least three times in duplicate and values were expressed as means ± standard deviation (SD). An unpaired student t-test was performed and a *p* values ≤ 0:05 were regarded as significant. For more than 2 groups, the data were analyzed using GraphPad Prism software. Comparisons between groups were performed using one-way ANOVA and Turkey’s Multiple Comparison Post Test. Differences were considered significant at p ≤ 0.05.

## References

[1] Kaushik A, Jayant RD, Bhardwaj V, Nair M (2018). Personalized nanomedicine for CNS diseases. Drug Discovery Today.

[2] Nair M, Jayant RD, Kaushik A, Sagar V (2016). Getting into the brain: Potential of nanotechnology in the management of NeuroAIDS. Advanced drug delivery reviews.

[3] Kaushik, A., Jayant, R. D. & Nair, M. Nanomedicine for neuroHIV/AIDS management. *Nanomedicine*, 10.2217/nnm-2018-0005 (2018).

[4] Kaminski, R. *et al*. Elimination of HIV-1 Genomes from Human T-lymphoid Cells by CRISPR/Cas9 Gene Editing. *Scientific reports* **6** (2016). 10.1038/srep22555 PMC4778041 26939770

[5] Zhang, Y. *et al*. CRISPR/gRNA-directed synergistic activation mediator (SAM) induces specific, persistent and robust reactivation of the HIV-1 latent reservoirs. *Scientific reports* **5** (2015). 10.1038/srep16277 PMC4633726 26538064

[6] Sagar V, Pilakka-Kanthikeel S, Pottathil R, Saxena SK, Nair M (2014). Towards nanomedicines for neuroAIDS. Reviews in medical virology.

[7] Deeks SG (2012). Towards an HIV cure: a global scientific strategy. Nature reviews Immunology.

[8] Kaushik A, Jayant RD, Sagar V, Nair M (2014). The potential of magneto-electric nanocarriers for drug delivery. Expert opinion on drug delivery.

[9] Ruiz A, Nair M, Kaushik A (2015). Recent update in NanoCure of NeuroAIDS. Science Letters Journal.

[10] Jayant R (2015). Sustained-release nanoART formulation for the treatment of neuroAIDS. International Journal of Nanomedicine.

[11] Nair M (2013). Externally controlled on-demand release of anti-HIV drug using magneto-electric nanoparticles as carriers. Nature communications.

[12] Guduru, R., Liang, P., Runowicz, C., Atluri, V. & Khizroev, S. Magneto-electric Nanoparticles to Enable Field-controlled High-Specificity Drug Delivery to Eradicate Ovarian Cancer Cells. *Nature Scientific Reports* **3** (2013). 10.1038/srep02953 PMC3797424 24129652

[13] Kaushik, A. K., Jayant, R. D. & Nair, M. *Advances in Personalized Nanotherapeutics*. (Springer, 2017).

[14] Vashist, A., Kaushik, A. K., Ahmad, S. & Nair, M. *Nanogels for Biomedical Applications*. Vol. 30 (Royal Society of Chemistry, 2017).

[15] Kaushik A, Nair M (2018). Nanotheranostic, Next Generation Prerequisite for Better Health. Journal of Nanotheranostics.

[16] Kaushik AEA (2016). Magnetically guided central nervous system delivery and toxicity evaluation of magneto-electric nanocarriers. Sci. Rep..

[17] Khalili K, Kaminski R, Gordon J, Cosentino L, Hu W (2015). Genome editing strategies: potential tools for eradicating HIV-1/AIDS. Journal of neurovirology.

[18] White, M. K., Hu, W. & Khalili, K. The CRISPR/Cas9 Genome Editing Methodology as a Weapon Against Human Viruses. *Discovery medicine* (2015). PMC4445958 25977188

[19] Liao, H. -K. *et al*. Use of the CRISPR/Cas9 system as an intracellular defense against HIV-1 infection in human cells. *Nature communications* **6** (2015). 10.1038/ncomms7413 25752527

[20] Mali P (2013). RNA-guided human genome engineering via Cas9. Science.

[21] Pellagatti, A., Dolatshad, H., Valletta, S. & Boultwood, J. Application of CRISPR/Cas9 genome editing to the study and treatment of disease. *Archives of toxicology*, 1–12 (2015). 10.1007/s00204-015-1504-y 25827103

[22] Xiao-Jie L, Hui-Ying X, Zun-Ping K, Jin-Lian C, Li-Juan J (2015). CRISPR-Cas9: a new and promising player in gene therapy. Journal of medical genetics.

[23] Zhang, F., Wen, Y. & Guo, X. CRISPR/Cas9 for genome editing: progress, implications and challenges. *Human molecular genetics*, ddu125 (2014). 10.1093/hmg/ddu125 24651067

[24] Sun W (2015). Self‐Assembled DNA Nanoclews for the Efficient Delivery of CRISPR–Cas9 for Genome Editing. Angewandte Chemie.

[25] Lee B (2018). Nanoparticle delivery of CRISPR into the brain rescues a mouse model of fragile X syndrome from exaggerated repetitive behaviours. Nature Biomedical Engineering.

[26] Alvarez-Carbonell D (2017). Toll-like receptor 3 activation selectively reverses HIV latency in microglial cells. Retrovirology.

[27] Kaushik A (2017). Investigation of ac-magnetic field stimulated nanoelectroporation of magneto-electric nano-drug-carrier inside CNScells. Scientific reports.

[28] Gorelik V, Krylov A, Sverbil V (2014). Local Raman spectroscopy ofDNA. Bulletin of the Lebedev Physics Institute.

[29] Barhoumi A, Zhang D, Tam F, Halas NJ (2008). Surface-enhanced Raman spectroscopy of DNA. Journal of the American Chemical Society.

[30] Thomas GJ (1999). Raman spectroscopy of protein and nucleic acid assemblies. Annual review of biophysics and biomolecular structure.

[31] Gajović A (2013). Temperature‐dependent Raman spectroscopy of BaTiO3 nanorods synthesized by using a template‐assisted sol–gel procedure. Journal of Raman Spectroscopy.

[32] Soler M (2004). Structural stability study of cobalt ferrite-based nanoparticle using micro Raman spectroscopy. Journal of magnetism and magnetic materials.

[33] Cheng Y, Morshed RA, Auffinger B, Tobias AL, Lesniak MS (2014). Multifunctional nanoparticles for brain tumor imaging and therapy. Advanced drug delivery reviews.

[34] Liu L (2008). Polymeric micelles anchored with TAT for delivery of antibiotics across the blood–brain barrier. Peptide Science.

[35] Malam Y, Loizidou M, Seifalian AM (2009). Liposomes and nanoparticles: nanosized vehicles for drug delivery in cancer. Trends in pharmacological sciences.

[36] Bourzac K (2012). Nanotechnology: Carrying drugs. Nature.

[37] Pardridge WM (2007). Blood–brain barrier delivery. Drug Discovery Today.

[38] Hu W (2014). RNA-directed gene editing specifically eradicates latent and prevents new HIV-1infection. Proceedings of the National Academy of Sciences.

[39] Persidsky Y (1999). Microglial and astrocyte chemokines regulate monocyte migration through the blood-brain barrier in human immunodeficiency virus-1 encephalitis. The American journal of pathology.

[40] Kanmogne GD (2007). HIV-1gp120 compromises blood–brain barrier integrity and enhance monocyte migration across blood–brain barrier: implication for viral neuropathogenesis. Journal of Cerebral Blood Flow & Metabolism.

[41] Yndart, A. *et al*. Investigation of Neuropathogenesis in HIV-1 Clade B & C Infection Associated with IL-33 and ST2Regulation. *ACS chemical neuroscience* (2015). 10.1021/acschemneuro.5b00156 26110635

